# Supplementary material for: Cobamide-producing microbes as a model for understanding general nutritional interdependencies in soil food webs
Source: Nat Commun. 2026 Jan 13;17:1533. doi: 10.1038/s41467-025-68255-6 (PMC12891699; doi:10.1038/s41467-025-68255-6)
Supplement: Supplementary file 1 — Supplementary Information [file 41467_2025_68255_MOESM1_ESM.pdf]

Supplementary Materials for

Cobamide-producing microbes as a model for understanding general nutritional interdependencies in soil food webs

Qi Zhang[1,2], Bingfeng Chen[3], Zhenyan Zhang[1,2], Yitian Yu[3], Mingkang Jin[4], Tao Lu[3], Ziyao Zhang[3], Qian Pang[3], Nuohan Xu[1,2], Jianqiang Sun[3], Jun Chen[5], Jichen Wang[4], Dong Zhu[6,*], Haifeng Qian[1,3,*] Josep Penuelas[7,8], Yong-Guan Zhu[4,6]

1. Institute for Advanced Study, Shaoxing University, Shaoxing 312000, P. R. of China
2. College of Chemistry & Chemical Engineering, Shaoxing University, Shaoxing 312000, P. R. of China
3. College of Environment, Zhejiang University of Technology, Hangzhou 310032, P. R. of China
4. State Key Laboratory of Urban and Regional Ecology, Research Center for Eco-environmental Sciences, Chinese Academy of Sciences, Beijing 100085, P. R. of China
5. Laboratory of Pollution Exposure and Health Intervention Technology, Interdisciplinary Research Academy, Zhejiang Shuren University, Hangzhou, 310021, P. R. of China
6. Key Laboratory of Urban Environment and Health, Institute of Urban Environment, Chinese Academy of Sciences, Xiamen 361021, P. R. of China
7. CSIC, Global Ecology Unit CREAF-CSIC-UAB, Bellaterra, Barcelona 08193, Catalonia, Spain
8. CREAF, Campus Universitat Autònoma de Barcelona, Cerdanyola del Vallès, Barcelona 08193, Catalonia, Spain

[*]Correspondence to: Dong Zhu (dzhu@iue.ac.cn) and Haifeng Qian (hfqian@zjut.edu.cn)

Supplementary Methods

Metadata analysis

Four datasets were collected: representative soil animal genomes, large-scale metagenomes, representative bacterial genomes, and 16S rRNA amplicon sequencing data.

To compile the largest possible set of representative soil fauna genomes as of June 3, 2023, we systematically searched for the taxonomic names of common soil invertebrates in the National Center for Biotechnology Information (NCBI) genome browser. This search yielded basic information, including species names, accession numbers, release dates, and assembly levels (e.g., contig and scaffold). Genomes were downloaded for further analysis. For species with multiple genome sequences, we selected the genome with publicly available annotations, the highest assembly level, and the latest release date. In total, 132 soil invertebrates representing 19 orders were included and subjected to quality control to ensure that all the genomes had > 95% completeness (Supplementary Data 9). We employed a hidden Markov model-based search to retrieve 954 BUSCOs from 65 reference metazoan genomes using OrthoDB (v.10; metazoa_odb10) [1].

In January 2022, we systematically retrieved large-scale soil metagenomic datasets from the European Nucleotide Archive (ENA; https://www.ebi.ac.uk/ena) using the following search queries: “soil AND metagenome,” “soil AND microbiome,” “farmland AND metagenome,” “soil AND metagenomic,” “land AND metagenome OR metagenomic,” and “soil metagenome OR metagenomic.” Samples were excluded based on the following criteria: (i) Exclusion of plant-associated environments (e.g.,

rhizosphere and phyllosphere) to minimize plant-derived metabolic interference with soil microbial profiling; (ii) absence of critical metadata fields (geolocation, habitat classification, sequencing platform, and project ID); (iii) omission of non-shotgun metagenomic datasets, including amplicon sequencing (16S/18S/ITS), meta-transcriptomes, or sequences generated from non-Illumina platforms (e.g., PacBio and Nanopore); and (iv) removal of metagenomes with average read lengths < 100 bp to ensure assembly feasibility (N50 >10 kb threshold). Ultimately, we collected 2,727 soil metagenomes from 24 countries across six continents for further analysis (Supplementary Data 2). Additionally, we queried the NCBI database in March 2022 with the terms "bacteria" [Organism], "soil" [Source], AND "genome" [Title], collecting 5,363 representative bacterial genomes derived from soil (Supplementary Data 10). We also gathered 40,039 high-quality MAG bins (the SMAG catalogue) from 3,304 soil metagenomes, as reported by Ma et al. [2].

To determine the distribution and functional spectrum of soil cobamide-producing microbes across human and animal guts worldwide, we searched Google Scholar and the Sequence Read Archive database of NCBI for metadata on June 10, 2023, using the terms "gut microbiota," "gut microbial community," "gut microbiome," and "gut bacteria." Next, we manually screened and excluded these samples based on the following criteria: (i) data derived from human skin and bile or animal-associated terrestrial or aquatic environments; (ii) data from patients treated with drugs, pathogens, probiotics, diet, or other interventions (to avoid interference of biochemical factors on gut microbial communities); and (iii) the use of other sequencing technologies, such as

metagenomic or metatranscriptome sequencing. After filtration, we maintained the quantitative balance and representativeness of the data from the different animal types. We collected 20,932 16S rRNA amplicon sequencing datasets from the gut microbiomes of 19 mammals (including humans), seven birds, seven insects, one annelid, and two amphibian and reptile species across 150 locations and 31 countries on six continents (Supplementary Data 7).

These data were downloaded from the European Nucleotide Archive (https://ebi.ac.uk/ena/) and the NCBI Sequence Read Archive (https://ncbi.nlm.nih.gov/sra/).

Metagenomic assemblies and reconstruction of soil MAGs

Metagenomic paired sequences were filtered by adapters, short reads, and low-quality reads using Trimmomatic [3] with the default parameters to ensure the removal of contaminants and the retention of high-quality reads for accurate downstream analyses. Metagenomic assemblies were reconstructed using MEGAHIT (v.1.2.9), which is based on the metawrap assembly and binning commands. The reads were mapped to contigs via Bowtie 2 [4] (v.2.3.5.1) with default parameters, and the resulting alignment files were sorted and indexed using SAMtools (v.1.9) [5]. The sorted BAM file was used to bind the contigs using a combination of CONCOCT (v.1.0.0) [6], MaxBin 2.0 (v.2.2.6) [7], and metaBAT2 (v.2.12.1) [8] with the default parameters to identify the MAGs. During refinement, the completeness and contamination of all MAGs were estimated using the lineage-specific workflow in CheckM (v.1.0.11) [9] to generate MAGs with ≥ 50% completeness and < 10% contamination. Finally, we obtained a total of 7,874 medium-

quality MAGs from all the soil metagenomic data (Supplementary Data 1).

Construction of the SCP (v.1.0) database

In accordance with our previous study [10], we applied hidden Markov model (HMM) profiles for genes essential to cobamide biosynthetic pathways, as defined by the Kyoto Encyclopedia of Genes and Genomes (KEGG) [11], TIGRFAM [12], PFAM [13] databases, and supplemented by the UniProtKB reference proteomes. We then employed the HMM search (v3.3.2) [14] with custom profiles to identify the corresponding genes within the predicted proteins of each MAG. To balance the sensitivity and specificity, an E-value threshold of 1×10^{-6} was applied during sequence scanning for each MAG [10,15,16].

Following Shelton's modified criteria [17], we categorized MAGs into four cobamide biosynthesis phenotypes based on the presence of key biosynthetic genes (Supplementary Data 11), implemented as follows: (1) 'very-likely producers' were defined as MAGs containing either all 25 anaerobic or all 23 aerobic pathway steps; (2) 'likely producers' required ≥ 4/5 tetrapyrrole precursor biosynthesis steps plus either ≥ 90% of anaerobic or aerobic pathway steps; (3) 'possible producers' met one of four intermediate thresholds: (i) ≥ 6/10 aerobic corrin ring steps plus ≥ 16/23 total aerobic steps, (ii) ≥ 9/12 anaerobic corrin ring steps plus ≥ 18/25 total anaerobic steps, (iii) ≥ 16/21 combined steps (tetrapyrrole+corrin ring+modification) for aerobic pathways, or (iv) ≥ 18/23 such combined steps for anaerobic pathways; and (4) 'non-producers' lacked sufficient evidence for any producer category. A total of 18,402 soil MAGs were identified as potential cobamide-producing microbes, respectively. Their taxonomic classification was further refined using the Genome Taxonomy Database Toolkit

(GTDB-Tk; v.1.7.0) [18] with 'classify_wf' function and r202 database. Notably, the identification of these four phenotypes was not strict because of the inherent issues of incompleteness and potential contamination in MAGs. To address this limitation, we improved bioinformatic approach to more accurately identify cobamide-producing microbes: 1) the sequence length of the cobamide-related contig should be ≤ 10 kbp; 2) the taxonomic information annotation results of the cobamide-related contig should be consistent with the taxonomic information annotation results of the MAG at family level [19]. After filtering, 4,340 cobamide-producing MAGs were clustered into 1,853 species-level MAGs at a 95% similarity threshold via dRep (v.3.2.2), with the taxonomic information and genome sequences constructed as the SCP (v.1.0_meta) database. We annotated 4,340 soil cobamide-producing MAGs based on the Kyoto Encyclopedia of Genes and Genomes database via DIAMOND (v.2.1.4; e value ≤ 10^{-3}, identity: 70%, coverage: 60%) to determine their functional groups.

In total, 1,827 16S rRNA gene sequences from all the cobamide-producing MAGs were extracted using the 'nhmmer' function (part of HMMER 3) from Barrnap (v.0.9) [20] with default parameters. These sequences were dechimerized using VSEARCH with default parameters based on the rdp_gold database [21] and filtered for short sequences (< 1000 bp) and low-quality sequences using SeqKit2 [22]. We next cleaned duplications and low-complexity sequences via Prinseq with the command "prinseq-lite.pl" [23] and finally obtained 1,370 high-quality sequences were obtained. Next, we predicted the coverage of the hypervariable regions of these sequences using the "Biopython" [24] package in Python (v.3.1.0) and selected the sequences with 100% coverage of the V3-

V5 hypervariable regions often used for amplification of various environmental and host DNA samples (Supplementary Fig. 18). Finally, we aligned the 1,370 high-quality sequences with the SILVA_138.1_SSU Ref_NR99 database via BLAST with a threshold of > 97% identity and > 80% coverage and eliminated the redundant sequences using the "Biopython" [24] package. Accordingly, 565 high-quality non-redundancy 16S rRNA sequences were integrated into SCP (v.1.0_16S) database, and their taxonomic information corresponded to the SILVA_138.1_SSU Ref_NR99 database. Thus, we further constructed the SCP (v.1.0_16S) database for annotations of amplicon sequencing data (see Data and Code availability for details). Meanwhile, 39,536 16S rRNA gene sequences from all the cobamide non-producing MAGs were extracted, and we finally obtained a total of 2,379 high-quality non-redundant sequences after filtering with the above framework (cobamide non-producer database). Moreover, identification of cobamide-producing microbes and non-producers from the 16S rRNA amplicon sequencing data was based on BLASTn alignment [25] of representative amplicon sequences to our constructed databases. We enforced a 100% sequence identity requirement for the variable regions covered by the sequencing amplicons.

Field experimental design and data analysis

Detailed information on sampling procedures, soil animal isolation and identification, and microbial DNA extraction and sequencing, is described in our previous study [26]. Briefly, field sampling was conducted across six sites in China between October and November 2017, spanning latitudes from 24.9°N to 41.7°N and longitudes from

102.95°E to 123.72°E, thereby encompassing most of the country's climatic zones (Supplementary Data 9). These sites were strategically designed to capture the ecological diversity of soil ecosystems, encompassing a range of climatic conditions, soil types, and vegetation types, all of which are known to influence the composition and activity of soil fauna and associated gut microbiota. These factors were carefully considered, as they can significantly impact the microbial community structure, biodiversity, and ecological interactions. For example, sites were chosen to represent both temperate and subtropical climates, allowing us to gain insights into how soil fauna and microbes adapt to various environmental pressures, such as temperature, humidity, and seasonal variability. Additionally, the selected sites varied in terms of soil type (loamy, sandy, and clay-rich soils) and vegetation cover (forested, grassland, and agricultural areas). These variations are key factors that shape the microbial and faunal communities and provide a comprehensive understanding of soil ecosystem dynamics.

In total, we obtained 238 collembolan samples (approximately 3,600 individuals), 60 nematode (approximately 6,000 individuals), 62 potworm (approximately 1,500 individuals), 146 oribatid mite (approximately 4,000 individuals), 122 predatory mite (approximately 2,000 individuals), 50 earthworm (approximately 1,000 individuals), and 60 soil samples from all the sites that were analyzed for microbial communities and ^{15}N isotopic signatures. The soil fauna taxa selected for this study were strategically chosen based on two key rationales: (1) These organisms dominate soil invertebrate communities globally, collectively representing > 75% of soil fauna biomass in temperate ecosystems [27], and thus, serve as ecologically relevant models for cross-

trophic interactions; (2) sampling targeted organisms spanning four defined trophic levels—primary decomposers (e.g., earthworms and potworms), microbivores (e.g., nematodes and collembolans), secondary consumers (e.g., oribatid mites), and apex predators (e.g., predatory mites)—to enable systematic tracking of cobamide producer distribution through food webs.

The sampled sites exhibited considerable variation in soil faunal composition owing to differences in climate, soil type, and vegetation, all of which are known to influence the abundance and diversity of soil organisms. These variations across locations could result in differences in microbial community structure, including the distribution of cobamide-producing microbes. Given that soil fauna can serve as habitats or vectors for microbial colonization, the variations in faunal composition may lead to distinct interactions between cobamide-producing microbes and their host organisms across different sites. For instance, sites with higher populations of decomposers, such as collembolans or earthworms, may support a more diverse array of cobamide-producing microbes because of their roles in nutrient cycling and microbial enrichment. Conversely, sites characterized by different vegetation types or soil properties may favor certain microbial taxa over others, resulting in site-specific patterns of cobamide producer distribution. This highlights the importance of ecological context in shaping the interactions between soil fauna and microbial communities. The dominant soil fauna at each sampling site were classified at the species level using morphological characteristics and DNA barcoding technology (for the universal primers; see the Supplementary Table 2).

The soil fauna samples, consisting of 30 nematodes, five Collembola, three potworms, five oribatid mites, five predatory mites, and three earthworms per sample, were surface sterilized through three washes with 0.5% sodium hypochlorite, followed by five rinses in sterile ultrapure water. The sterilized samples were subsequently transferred into sterile centrifuge tubes and homogenized using a microelectric tissue homogenizer. DNA was extracted from these faunal samples using a DNeasy Blood and Tissue Kit (QIAGEN, Germany), following the manufacturer's protocol. Each extraction yielded a minimum of 30 ng of DNA, with concentrations across different animal types normalized to 1 ng μL^{-1} before PCR amplification. All extracted DNA was stored at –20 °C until further analysis.

Laboratory experimental design

Soil moisture was maintained at 60% of the maximum soil moisture content (60.23%), as detailed in OECD guideline 220 (OECD, 2004). We added 90 g of soil to sterilized glass beakers (diameter, 5 cm; height, 7 cm) to establish the soil microcosms (n = 16). We then randomly selected 30 adult *E. crypticus* (33–40 days old) with visible gonads from the synchronized media and placed them into the established soil microcosms under controlled culture conditions (800 lux light, 8 h:16 h light–dark cycle, 18±2 °C, and 75% humidity) for 21 days. To mitigate the effects of feeding on the gut microbiota, we only maintained adequate water replenishment in the soil microcosm twice weekly during this period.

The initial microcosm soil and synchronized *E. crypticus* were sampled as day 0 soil and gut samples (n = 4 biological replicates), respectively. We subsequently collected

soil and gut samples from multiple microcosms on the day 2, 7, 14, and 21. First, 5 g of soil (excluding worms) and all adult *E. crypticus* were collected for further testing. Five freshly collected *E. crypticus* were immediately euthanized with chloroform to prevent continued feeding and excretion of the intestinal contents. Sacrificed *E. crypticus* were immersed in a 2% sodium hypochlorite solution for 10 s to remove surface microorganisms of *E. crypticus* and then rinsed five times with sterile water. To extract the DNA, we subsequently obtained the gut tissue of *E. crypticus* under a stereomicroscope (SMZ-168-TLED, China) on a sterile, clean bench. Additionally, the other 20 newly collected *E. crypticus* were washed five times with ultrapure water and then placed in ultrapure water for 2 h to expel as much of the gut contents as possible. These samples were then placed in a 2-mL centrifuge tube and frozen in liquid nitrogen for RNA extraction.

Determination of vitamin B_{12}

Cells of *B. megaterium* ATCC 14581 or *S. violaceus* NBC_00450 were pelleted by centrifugation (15,000 × g for 10 min at 4 °C), washed twice to remove extracellular metabolites, and resuspended in lysis buffer (50 mM Tris-HCl, 1 mM EDTA, 1% Triton X-100, and protease inhibitor cocktail). After 30-min ice incubation with vortexing every 5 min, lysates were centrifuged (20,000 × g for 15 min at 4 °C) to collect supernatants containing intracellular components. Vitamin B_{12} levels were quantified according to the instructions provided with the vitamin B_{12} ELISA Kit purchased from Shanghai Tongwei Biotechnology Co., Ltd. (Shanghai, China).

Design of the comparison experiment

We assessed the effects of *B. megaterium* ATCC 14581 and *S. violaceus* NBC_00450 on the growth of adult *E. crypticus*. Ninety synchronized adult *E. crypticus* (with obvious gonads) of similar body length were divided among nine Petri dishes. Each dish received 300 μL of *B. megaterium* ATCC 14581 solution (10^5 CFU/mL) or *S. violaceus* NBC_00450 solution (10^5 CFU/mL) for the treatments, or sterile water as the control. All the treatment groups consisted of three biological replicates, which were housed in an artificial climate box under the same conditions as mentioned previously. After an initial 2 days of colonization, we measured the body length of *E. crypticus* over the next 7 days.

To determine the optimal concentration of *B. megaterium* ATCC 14581 for colonization, we introduced mixed bacterial solutions with concentrations of 10^4, 10^5, 10^6, 10^7, and 10^8 CFU·mL^{-1} into Petri dishes that contained 50 synchronized *E. crypticus* strains and culture media under the following conditions: relative humidity, 75%; temperature, 18 °C; and light/dark photoperiod (800 lux), 16 h/8 h. The bacterial solutions were added once every 2 days for a total of three applications. After 7 days of colonization, we determined the number of adults and eggs using ImageJ software [28].

Design of the development rate experiment

First, we examined their effects on early developmental growth. Sixty synchronized eggs were evenly distributed into 12 Petri dishes, and each dish received 300 μL of *B. megaterium* ATCC 14581 suspension, Vitamin B_{12} solution, or sterile water (control), applied every two days for a total of three applications (n = 4 biological replicates per treatment). The dishes were maintained in an artificial climate chamber under

controlled conditions (800 lux light, 8 h:16 h light:dark cycle, 18 ± 2°C, and 75% relative humidity). Larvae hatched after 7 days of colonization, and the number of larvae as well as hatching success per egg were recorded over a 9-day period. Once the larvae reached the growth phase (15 days old), their body length was measured.

Next, we evaluated the effects of *B. megaterium* ATCC 14581 and Vitamin B_{12} on adult growth and reproduction. Four hundred synchronized adult *E. crypticus* (with visible gonads) were evenly allocated to eight Petri dishes. Each dish received 300 μL of *B. megaterium* suspension (treatment, n = 4), Vitamin B_{12} (treatment, n = 4), or sterile water (control, n = 4). After 2 days of colonization, we monitored egg production and measured adult body length over a 7-day period.

Third, to confirm the causal relationship between cobamide production capacity of cobamide-producing microbes and their host function, we determined the effects of *Bacillus megaterium* ATCC 14581 cultures supplemented with 0, 1, and 10 μM Co^{2+} (vitamin B_{12} production was 0.97 ± 0.23, 1.35 ± 0.25, and 2.29 ± 0.37 μg/L, respectively) on *E. crypticus* growth after 48 h. Ninety synchronized adult *E. crypticus* (with obvious gonads) of similar body length were divided into nine Petri dishes. Each dish received 300 μL of *B. megaterium* ATCC 14581 solution supplemented with 0, 1, or 10 μM Co^{2+} for the treatment. All the treatment groups consisted of three biological replicates, which were housed in artificial climate boxes under the same conditions as mentioned previously. After 2 days of colonization, we measured the body length of *E. crypticus* over 7 days.

Finally, we investigated the combined effects of *B. megaterium* ATCC 14581 and

Vitamin B_{12} on *E. crypticus* growth and gut microbiota. A total of 450 synchronized adult *E. crypticus* (with obvious gonads) of similar body lengths were divided into nine Petri dishes. Each dish received 300 μL of *B. megaterium* ATCC 14581 solution or Vitamin B_{12} (1 μg/L) for the treatments, or sterile water as the control. All the treatment groups consisted of three biological replicates, which were housed in an artificial climate box under the same conditions as mentioned previously. After 7 days, we recorded the body length of all adults and randomly selected five worms from each Petri dish for DNA extraction.

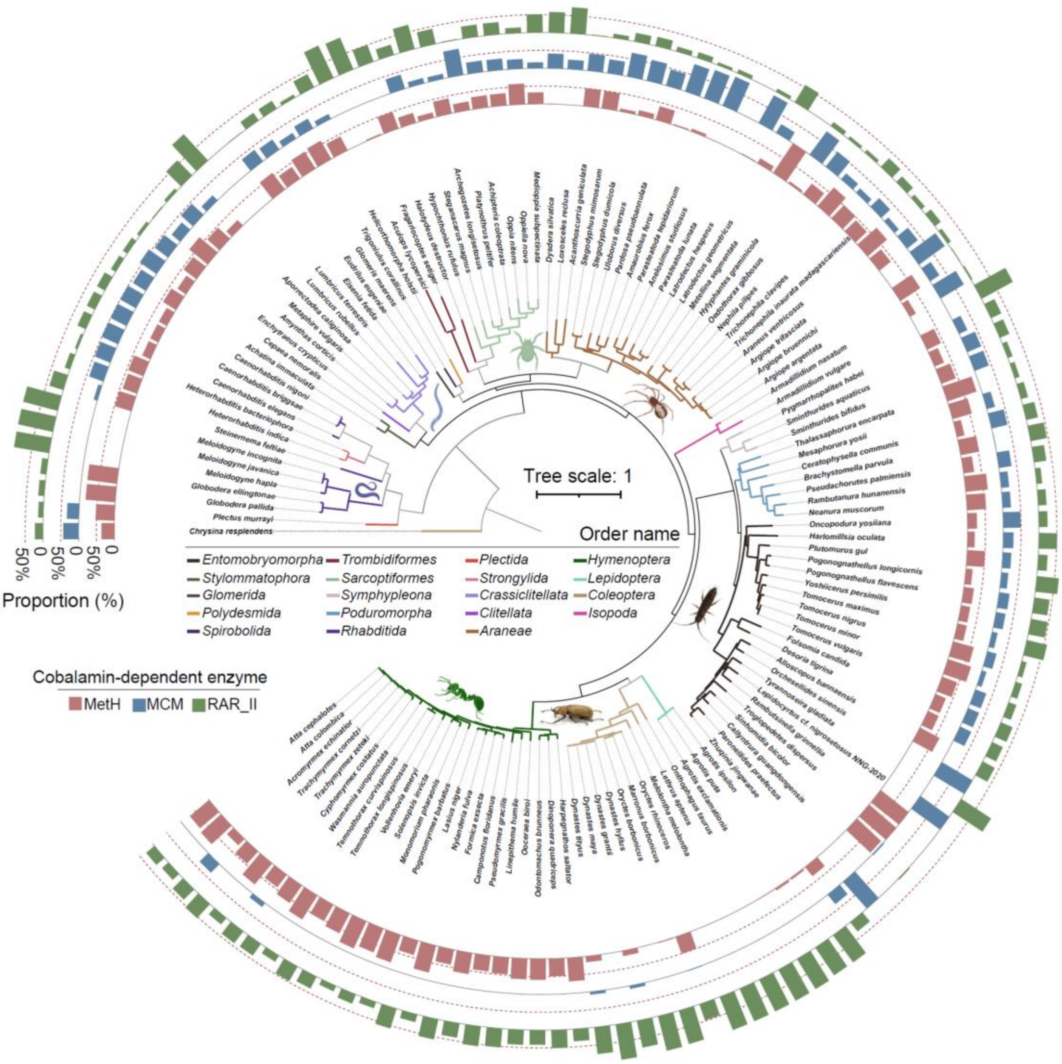

Supplementary Figure 1. Distribution of cobamide-dependent enzymes on the maximum likelihood phylogeny of 130 soil faunas. We sampled 130 soil faunas representing 19 orders from the National Center for Biotechnology Information Genome database. The phylogeny was a concatenated maximum-likelihood tree inferred from the 954 single-copy BUSCO genes analysis. Almost all the soil invertebrates (127/130) contained cobamide-dependent enzymes.

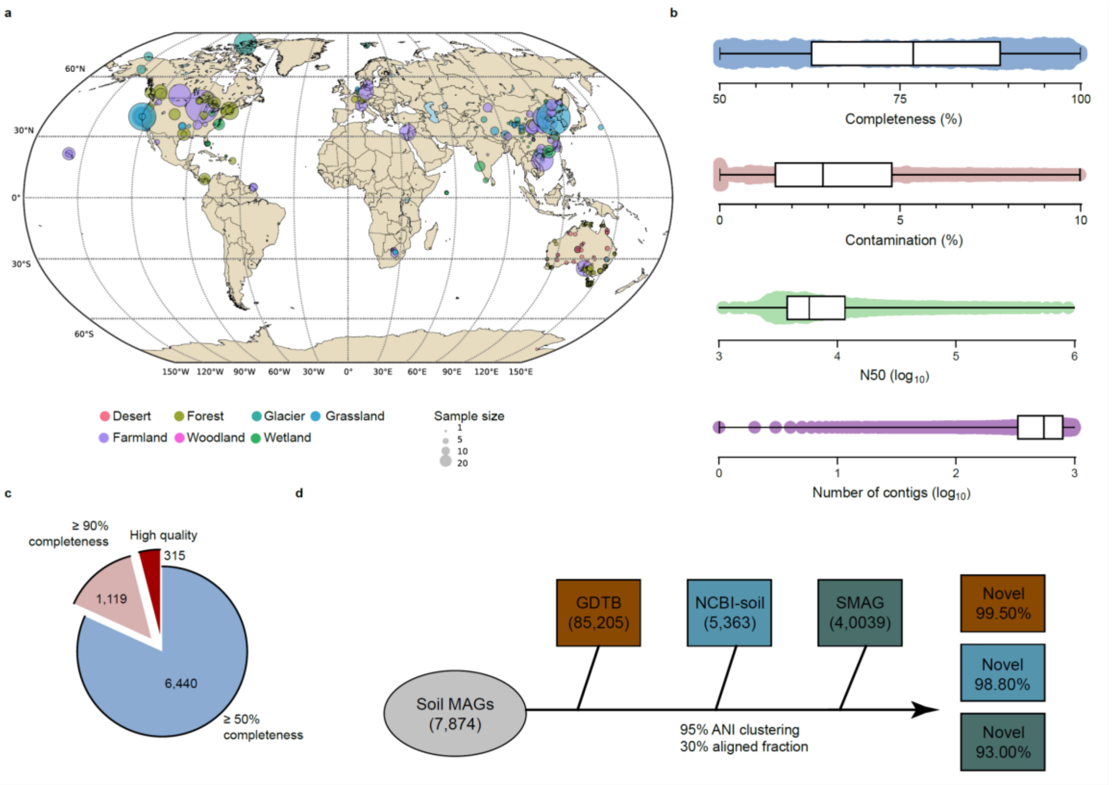

Supplementary Figure 2. Recovery of genomes from globally distributed soil metagenomes. a, Geographic distribution of recovered 7,874 MAGs from 2,727 soil metagenomes within various sub-habitats. **b,** Distribution of quality metrics across the soil MAGs. **c,** All MAGs have a completeness of at least 50% and a contamination level of less than 10%. **d,** Identification of the novelty of these soil MAGs. Representative genomes from Genome Taxonomy Database (85,205 genomes), National Center of Biotechnology Information (5,363 soil genomes), and SMAG (40,039 soil genomes) were then included in the clustering to identify the novelty of microbial species.

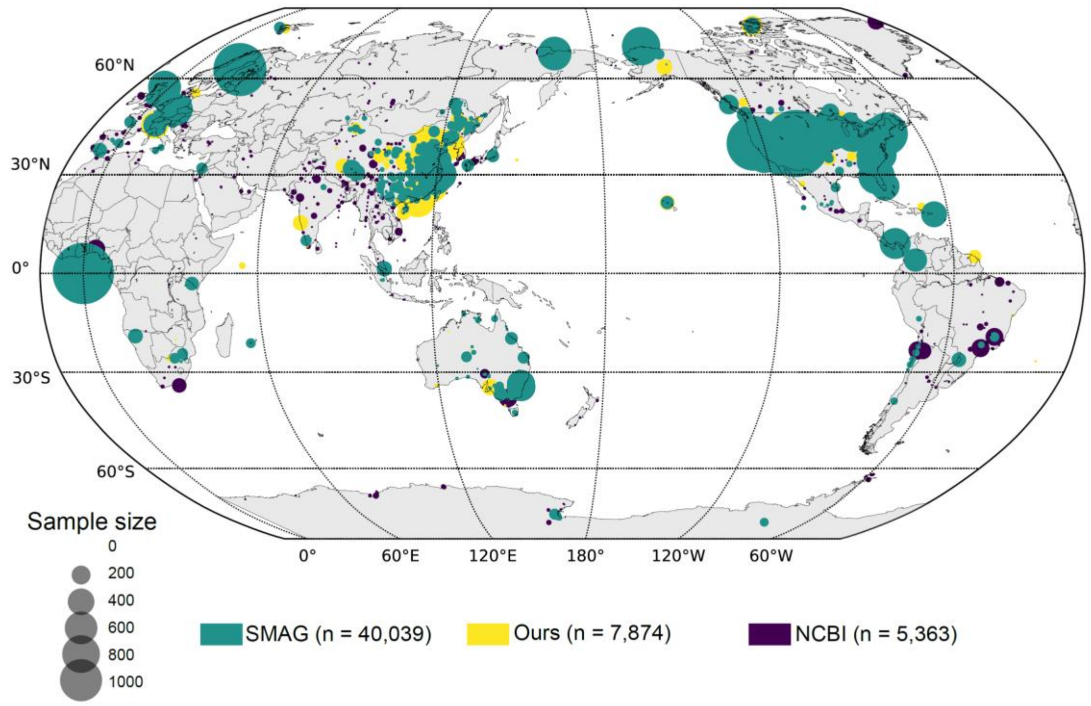

Supplementary Figure 3. Geographical distribution of metadata. Circle size indicates the number of soil genome samples. Green, yellow, and purple circles indicate ours, SMAG, and NCBI databases, respectively. For more details, see Dataset S1 and Dataset S2. Ours: Soil metagenome-assembled genomes recovered in this study; SMAG: Soil metagenome-assembled genome; NCBI: National Center of Biotechnology Information. Source data are provided as a Source Data file.

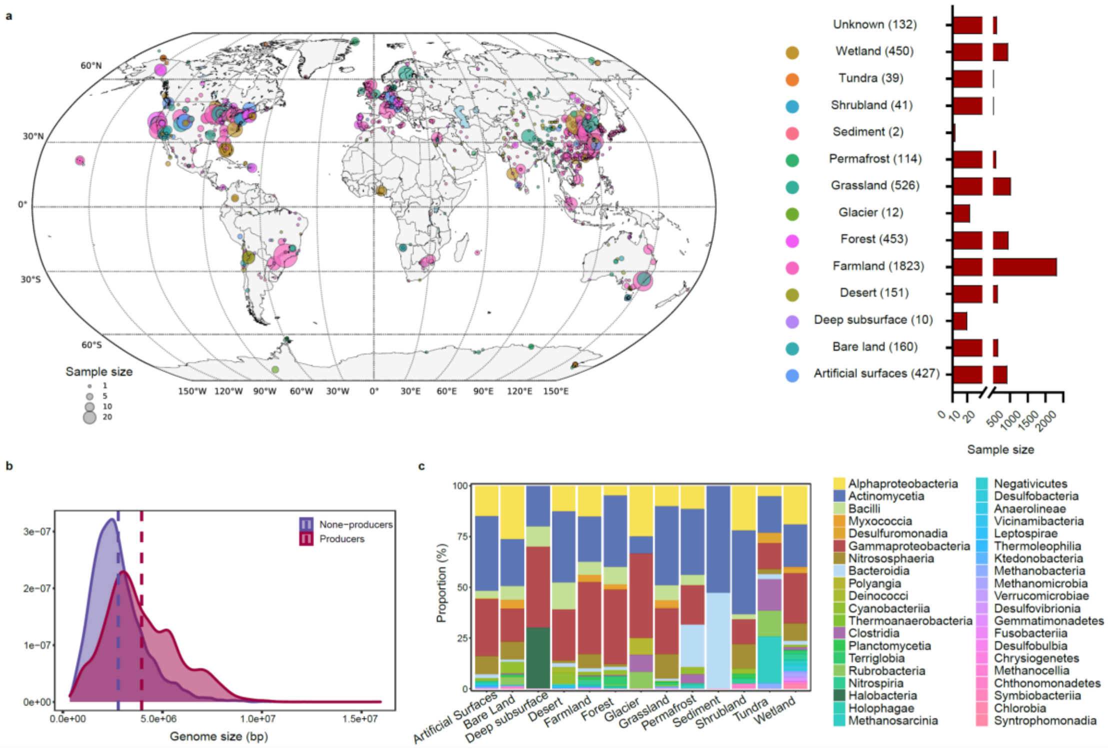

Supplementary Figure 4. Cobamide-producing microbes were habitat-specific across global terrestrial ecosystem. a, Global distribution of soil cobamide-producing microbes across diverse soil types. **b,** The genome size of cobamide-producing microbes was greatly higher than nonproducer (except cobamide dependents). Source data are provided as a Source Data file. **c,** The taxonomical composition of soil cobamide-producing taxa across diverse soil types. Source data are provided as a Source Data file.

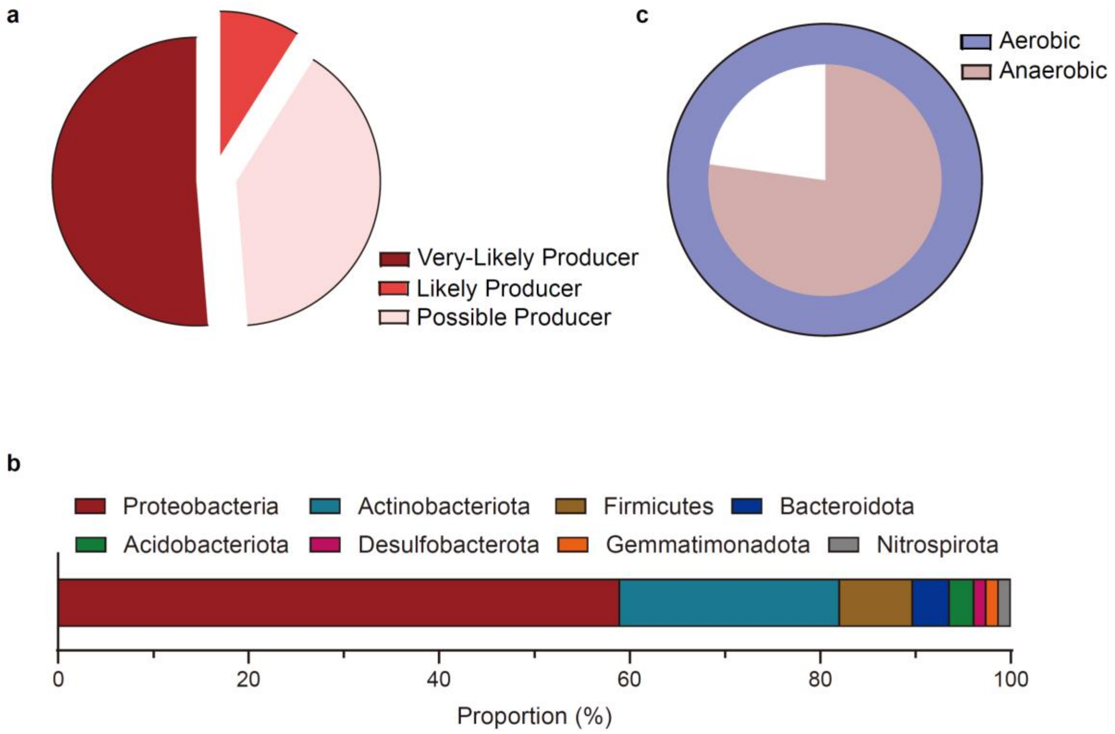

Supplementary Figure 5. Classification and composition of cobamide-producing microbes in the guts of soil fauna. a, Proportion of cobamide-producing microbes categorized by production likelihood: very-likely (dark red), likely (red), and possible producers (light pink). **b,** Taxonomic distribution of cobamide-producing microbes at the phylum level, with *Proteobacteria* being the most dominant group, followed by *Actinobacteriota*, *Firmicutes*, *Bacteroidota*, and others. **c,** The proportion of cobamide-producing microbes that undergo aerobic (blue) and anaerobic (pink) biosynthesis of cobamide.

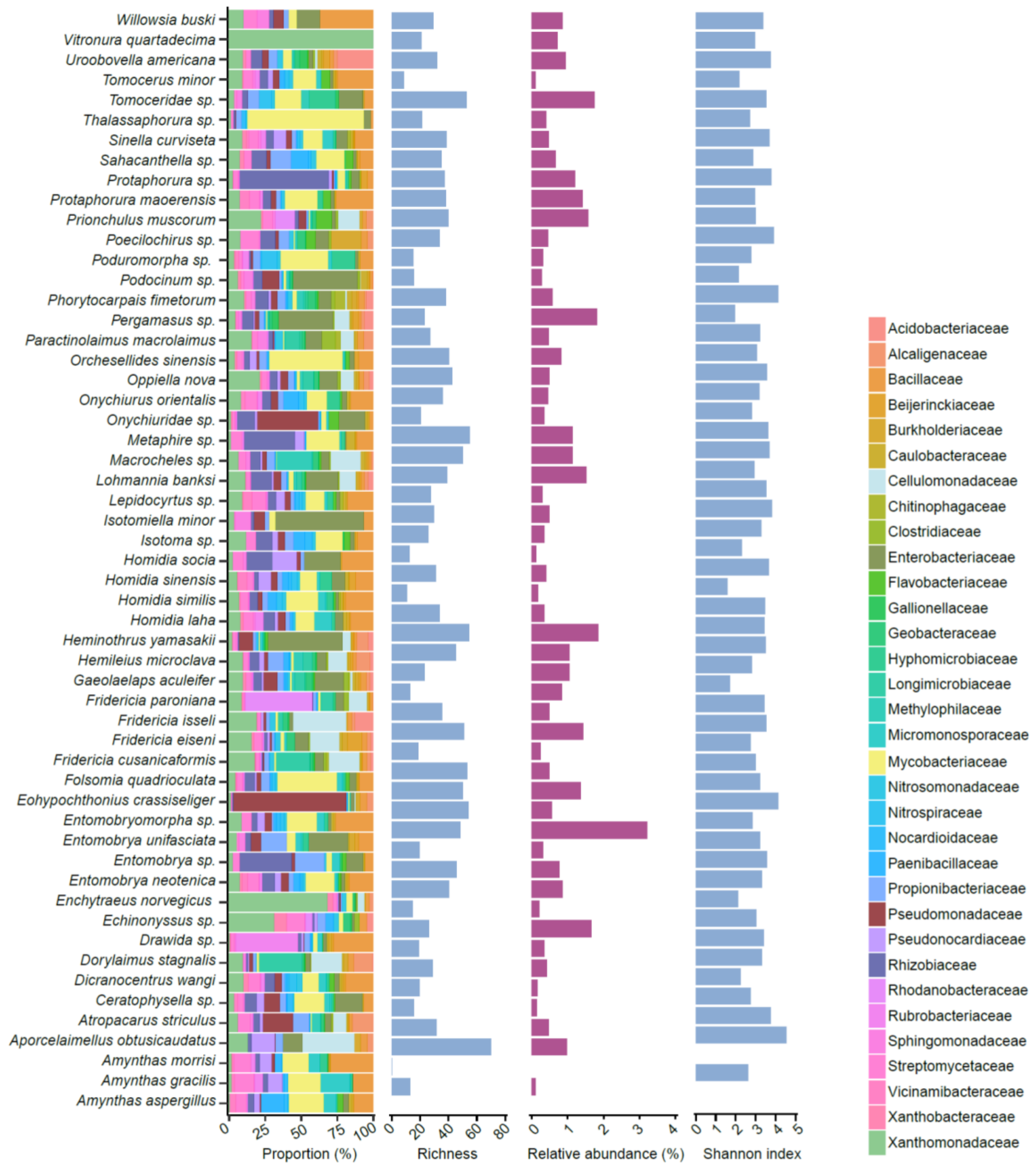

Supplementary Figure 6. Composition and diversity of cobamide-producing microbes in various soil fauna guts. The stacked bar plot on the left shows the relative proportion (%) of each bacterial family in the microbiome of different soil fauna species, with families represented by distinct colors as indicated in the legend on the right. The bar charts in the middle and right display species richness, relative abundance (%), and Shannon diversity index of cobamide-producing microbes across 55 different soil fauna species. Source data are provided as a Source Data file.

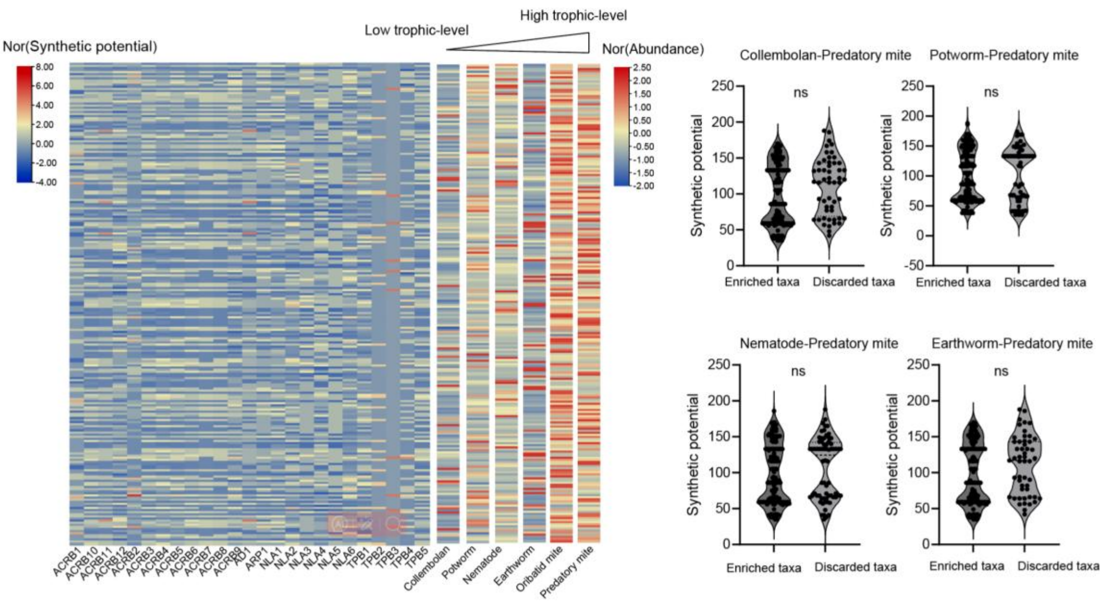

Supplementary Figure 7. The cobamide biosynthetic potential of cobamide-producing microbes across soil food web. The abundance and biosynthetic potential of cobamide-producing microbes across soil food web. The scale from blue to red indicates the value from high to low. The biosynthetic potential of enriched or discarded taxa between low trophic-level and top trophic-level soil fauna. "ns" indicates no significant difference between two groups (two-sided *t* test, $p > 0.05$). Specifically, each horizontal row represents one cobamide-producing taxon identified from the gut of soil fauna. The synthesis potential shown in the figure corresponds to the number of cobamide biosynthesis genes detected in the genome of cobamide-producing microbes. Source data are provided as a Source Data file.

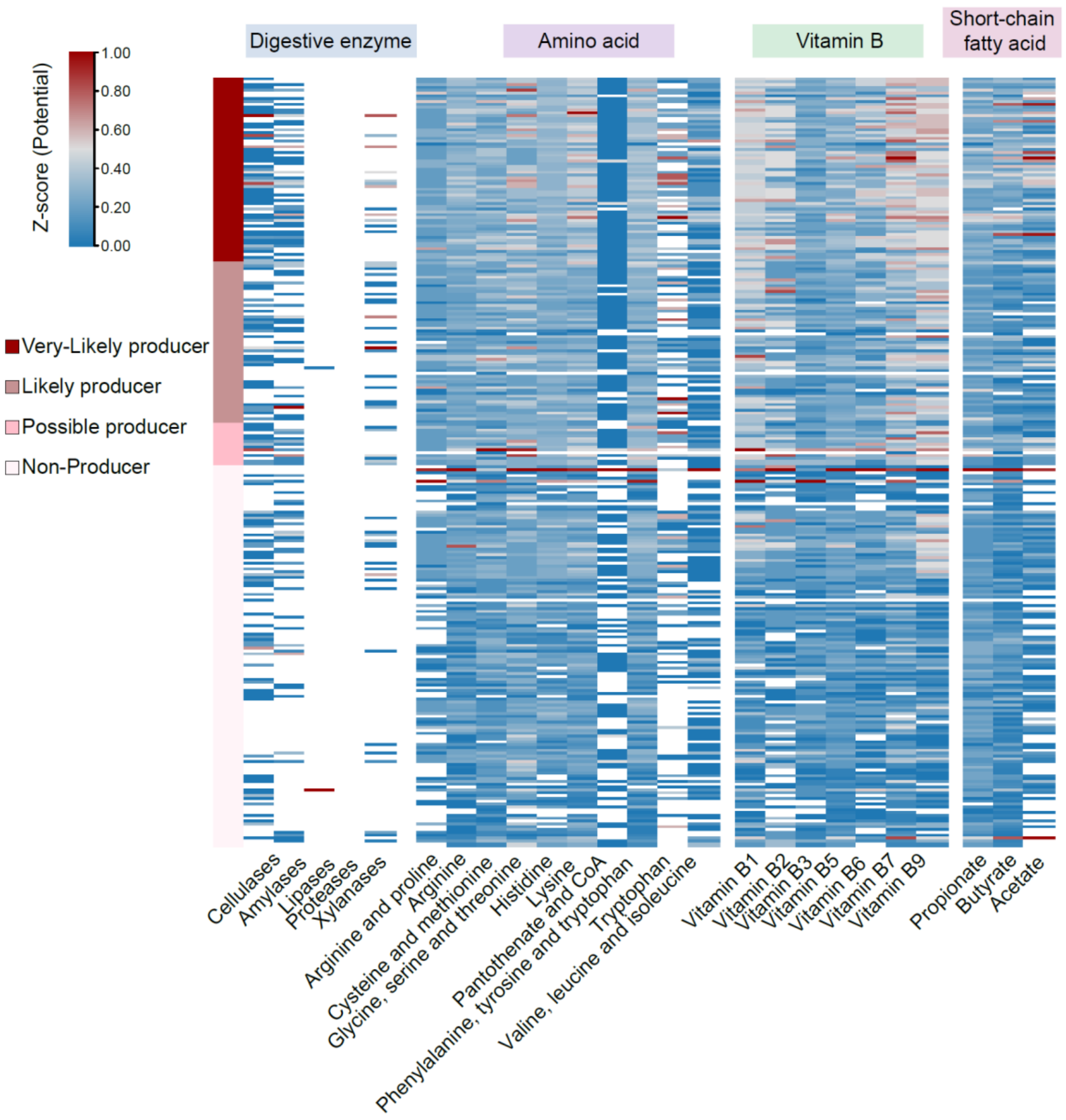

Supplementary Figure 8. Functional spectrum of cobamide-producing microbes detected in the guts of soil fauna. The biosynthesis potential of digestive enzymes, amino acid, vitamin B, and short-chain fatty acids in the soil cobamide-producing microbes detected in humans and animals. The color scale indicates the normalized counts (per genome) from low (blue) to high (red). Source data are provided as a Source Data file.

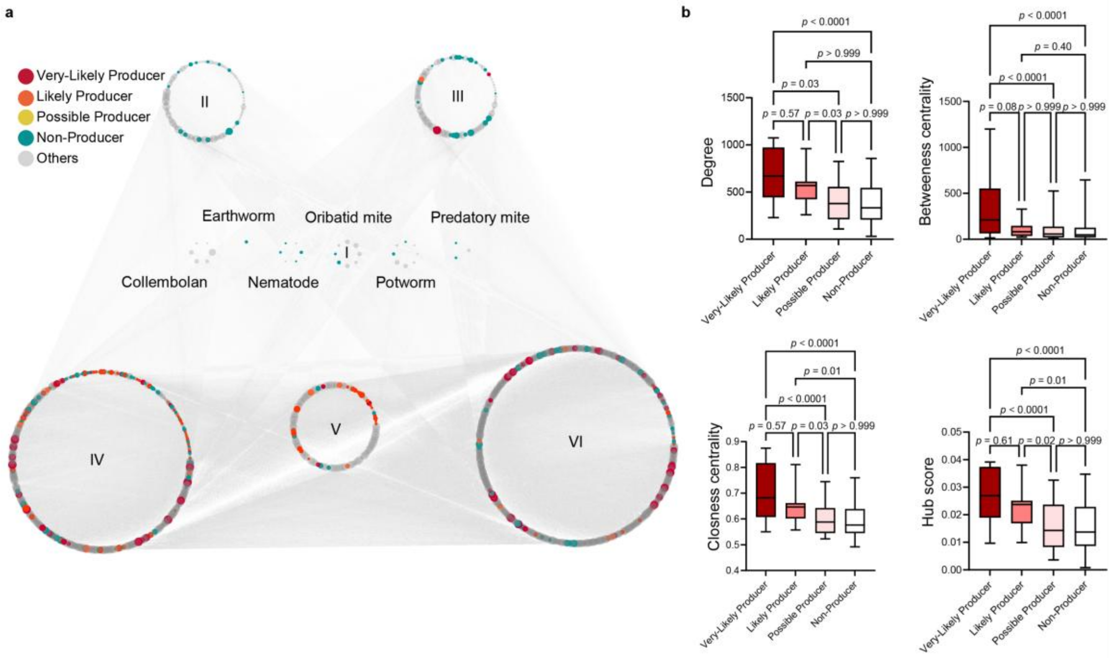

Supplementary Figure 9. The role of different phenotypes in the multi-trophic level co-occurrence network. a, The microbial co-occurrence network across multi-trophic level soil faunas. Different color circles indicate the classification of soil cobamide-producing bacteria. I-VI presents the trophic breadth of soil cobamide-producing bacteria colonized in fauna guts. **b,** Network topology data of different phenotypes in the multi-trophic level co-occurrence network. The position of cobamide-producing microbes in the co-occurrence network declines with the decrease in the biosynthetic potential of cobamide. Statistical significance was determined using two-sided Kruskal-Wallis test with Dunn's test. Source data are provided as a Source Data file.

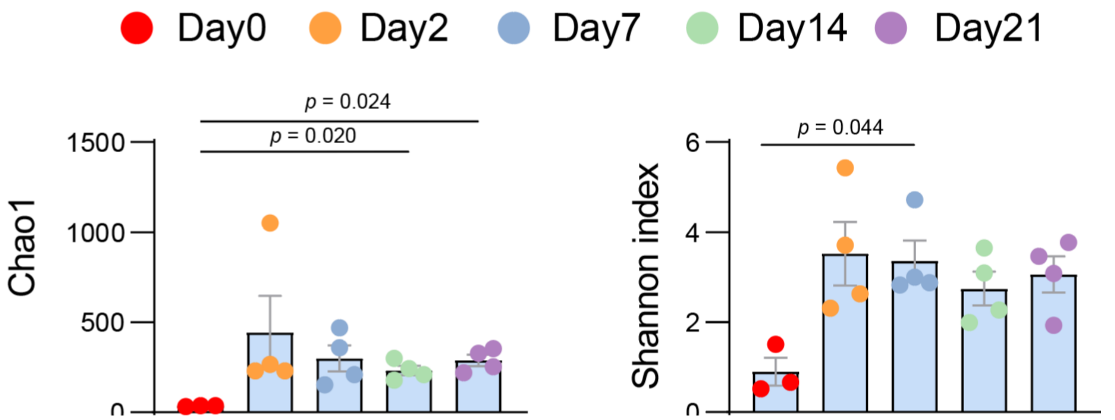

Supplementary Figure 10. The Shannon and Chao1 indices in the *E. crypticus* gut over time. The alpha diversity of *E. crypticus* gut bacterial community (Chao1 and Shannon indices). Different letters indicate significant differences among the groups, and the statistical significance is determined using two-sided one-way ANOVA with Tukey's multiple comparison test. Source data are provided as a Source Data file.

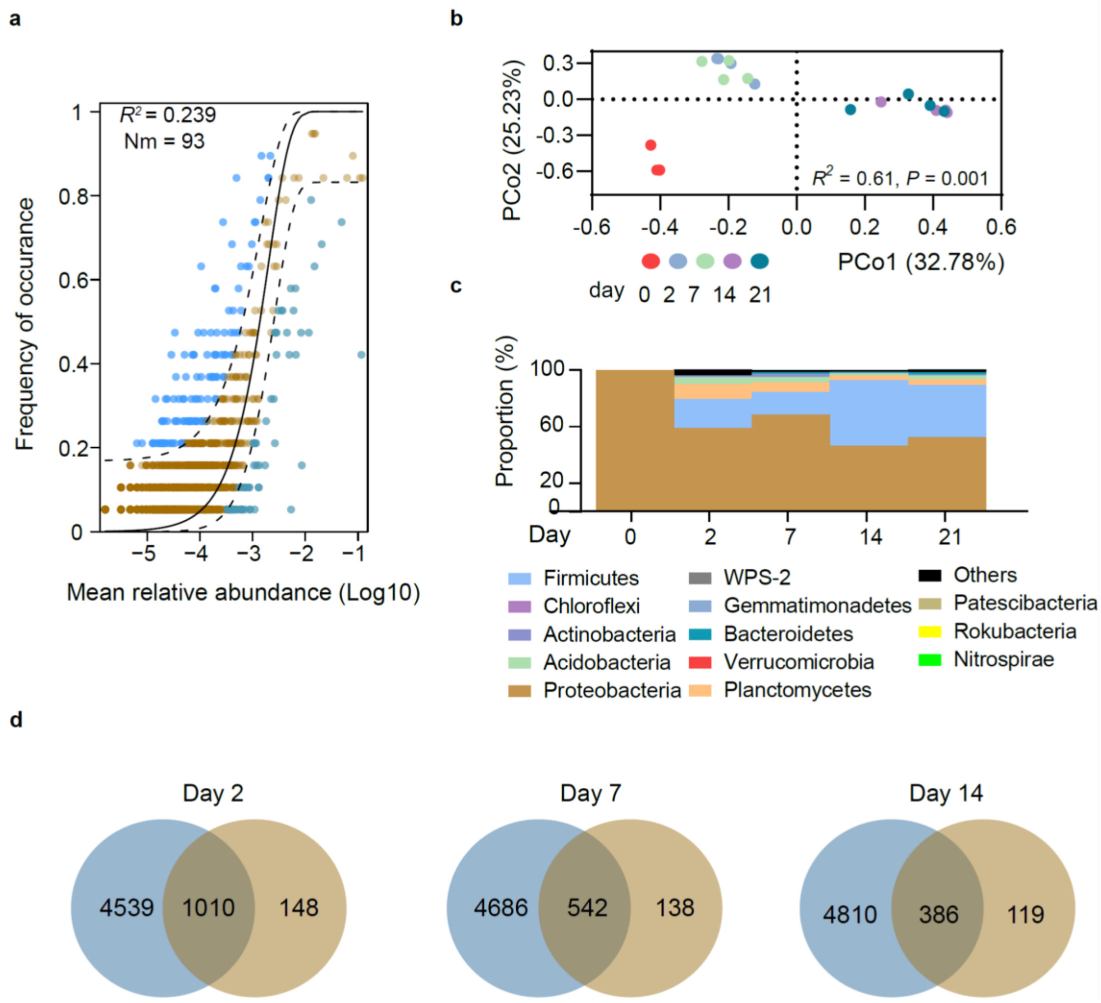

Supplementary Figure 11. Assembly, structure, and composition of the *E. crypticus* gut microbiome over time. a, Natural assembly patterns of *E. crypticus* gut microbial community in the soil ecosystem. Each point represents a microbial taxon, with color indicating different taxa. The solid line shows the logistic regression curve, with the dashed lines indicating the confidence interval (R^2 = 0.239, Nm = 93). **b,** Principal Coordinates Analysis of the gut microbiome in soil fauna across different days (Day 0, 2, 7, 14, and 21) based on Bray-Curtis dissimilarity. **c,** Taxonomic composition of soil fauna gut microbiome at the phylum level across different days. Each color represents a different microbial phylum, with changes in relative abundance observed over the course of the study. **d,** The shared and unique OTUs between soil (blue) and *E. crypticus* (brown) microbiomes during the microbial dynamic phase.

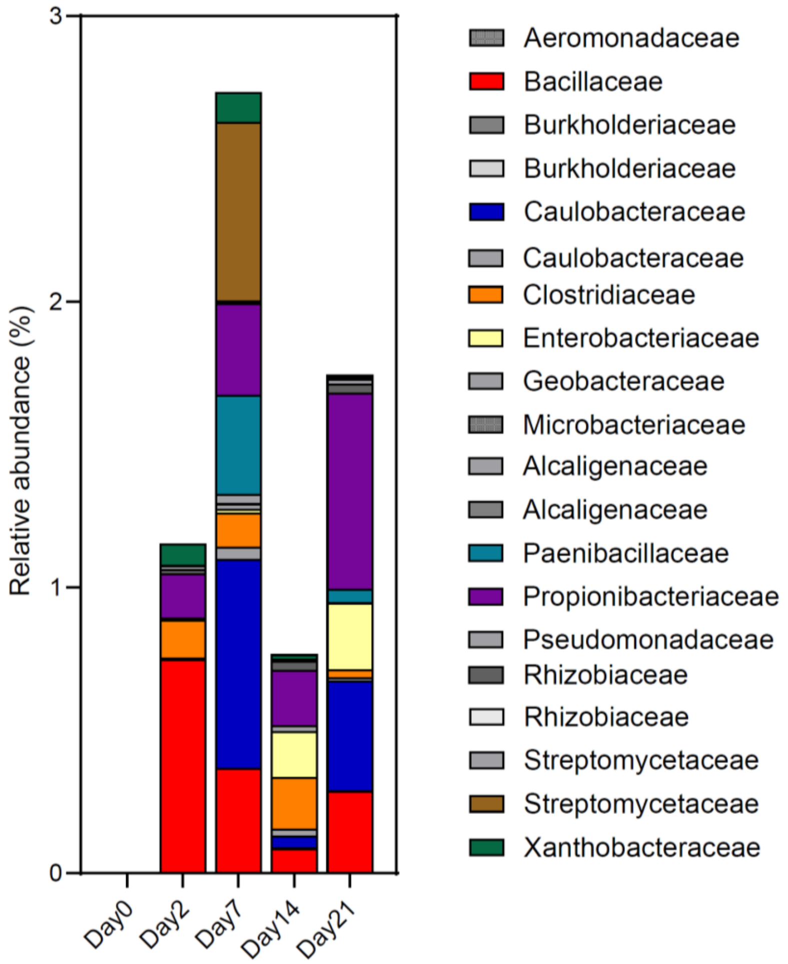

Supplementary Figure 12. Composition of cobamide-producing microbes in the gut of *E. crypticus* over time. With prolonged contact with soil microorganisms, some of these taxa lost their ecological niches, whereas others were replaced by transient taxa within the gut of the soil fauna. Source data are provided as a Source Data file.

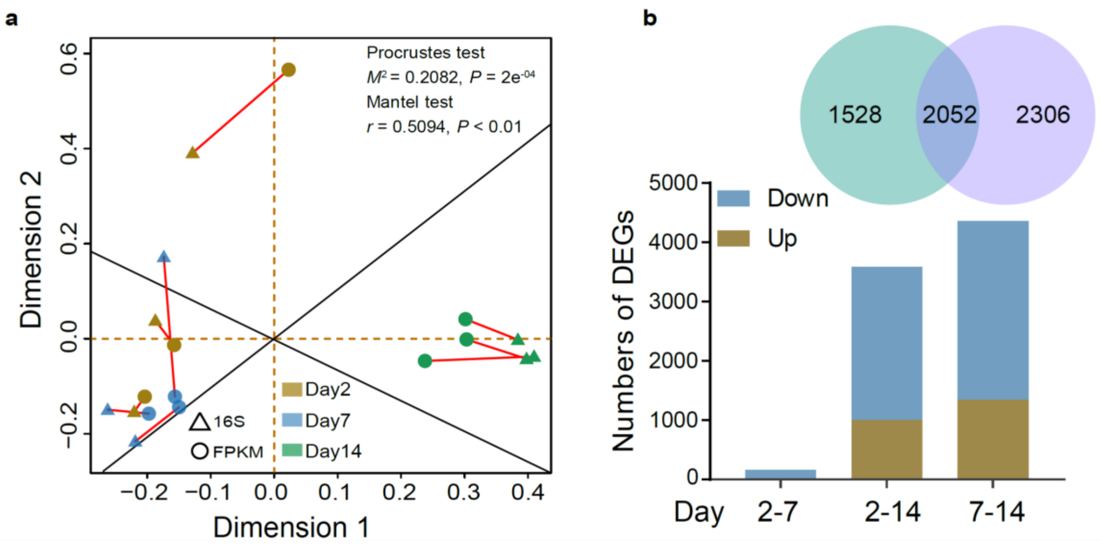

Supplementary Figure 13. Transcriptome of *E. crypticus* on Days 2-7. a, Procrustes analysis (M^2: Deviation sum of squares) and mantel test (r: correlation coefficient) showed a correlation between transcript gene expression values and soil-derived bacterial communities. **b,** Numbers of differentially expressed genes on days 2-7, 2-14 and 7-14. A Venn diagram illustrates the overlap and distinct differentially expressed genes when comparing the 2-day vs 14-day and 7-day vs 14-day experimental groups.

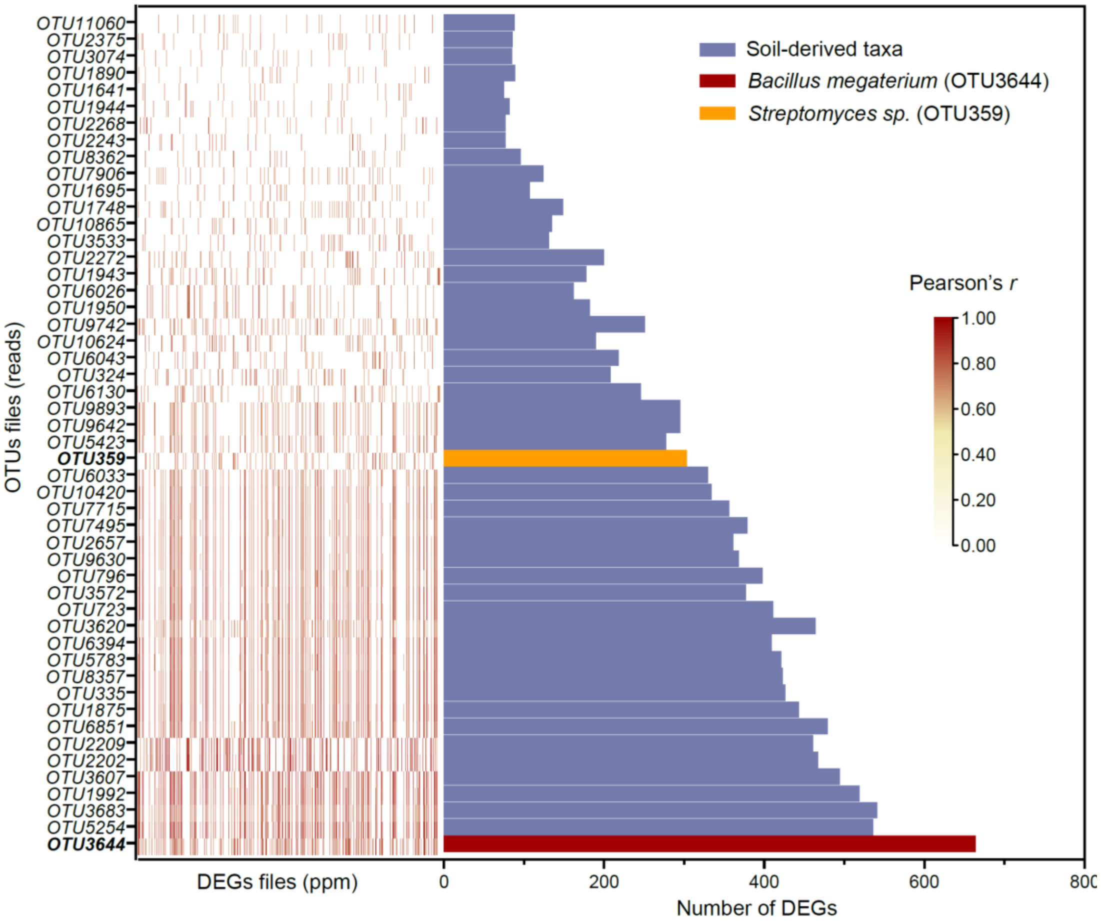

Supplementary Figure 14. Correlation between soil-derived OTUs and differentially expressed genes in *E. crypticus* during Days 2-7. *Bacillus megaterium* (OTU3644) and *Streptomyces sp.* (OTU359) showed stronger associations with host gene expression than other stable colonizing soil-derived taxa in the *E. crypticus* gut. Correlations between OTUs and differentially expressed genes were calculated using Pearson correlation analysis ($r > 0.6$, adjusted $P < 0.05$). This graph displays the top 50 OTUs most strongly associated with differentially expressed genes. Source data are provided as a Source Data file.

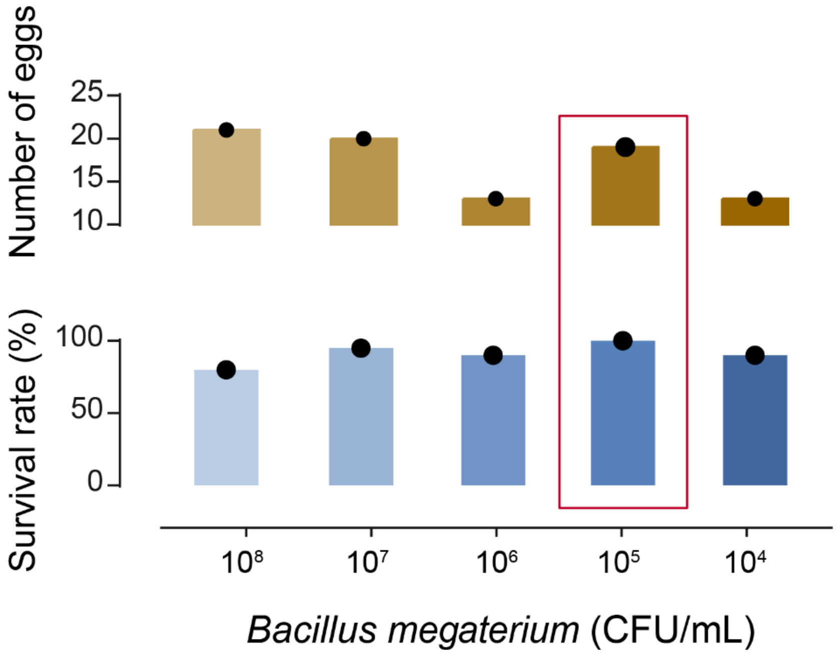

Supplementary Figure 15. The optimal artificial colonization concentration of *B. megatherium* ATCC 14581 for *E. crypticus*. Effects of different concentrations of *B. megatherium* ATCC 14581 on the survival and ovulation rate of *E. crypticus*. 10^5 CFU/mL was the optimal artificial colonization concentration for *E. crypticus*. Source data are provided as a Source Data file.

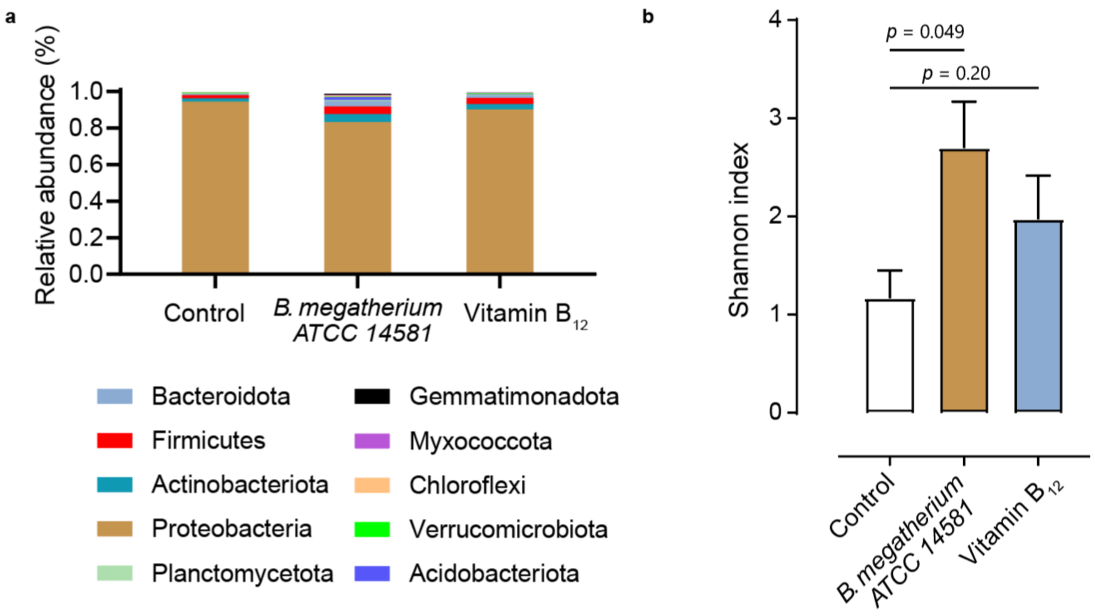

Supplementary Figure 16. The effect of *B. megatherium* ATCC 14581 and Vitamin B_{12} on *E. crypticus* gut microbiome. a, Oral supplementation of *B. megatherium* ATCC 14581 and Vitamin B_{12} altered the composition of the gut microbiome of *E. crypticus*. **b,** The Shannon index of gut microbial community in *E. crypticus* after treatment with *B. megatherium* ATCC 14581. "*" indicates significant difference between two groups (two-sided *t* test, $P < 0.05$). Source data are provided as a Source Data file.

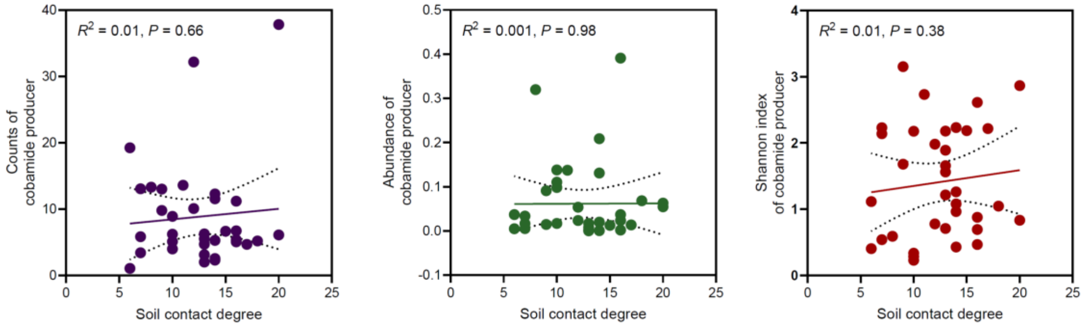

Supplementary Figure 17. The correlation between soil contact degree and cobamide-producing microbes across various animals. Ordinary least square linear regression shows that there was no correlation between the contact degree and the colonization of cobamide in the gut. The degree of soil contact was calculated based on five indicators: activity range, diet, activity time and frequency, dust exposure, and food web association within the soil environment. Source Data are provided as a Source data file.

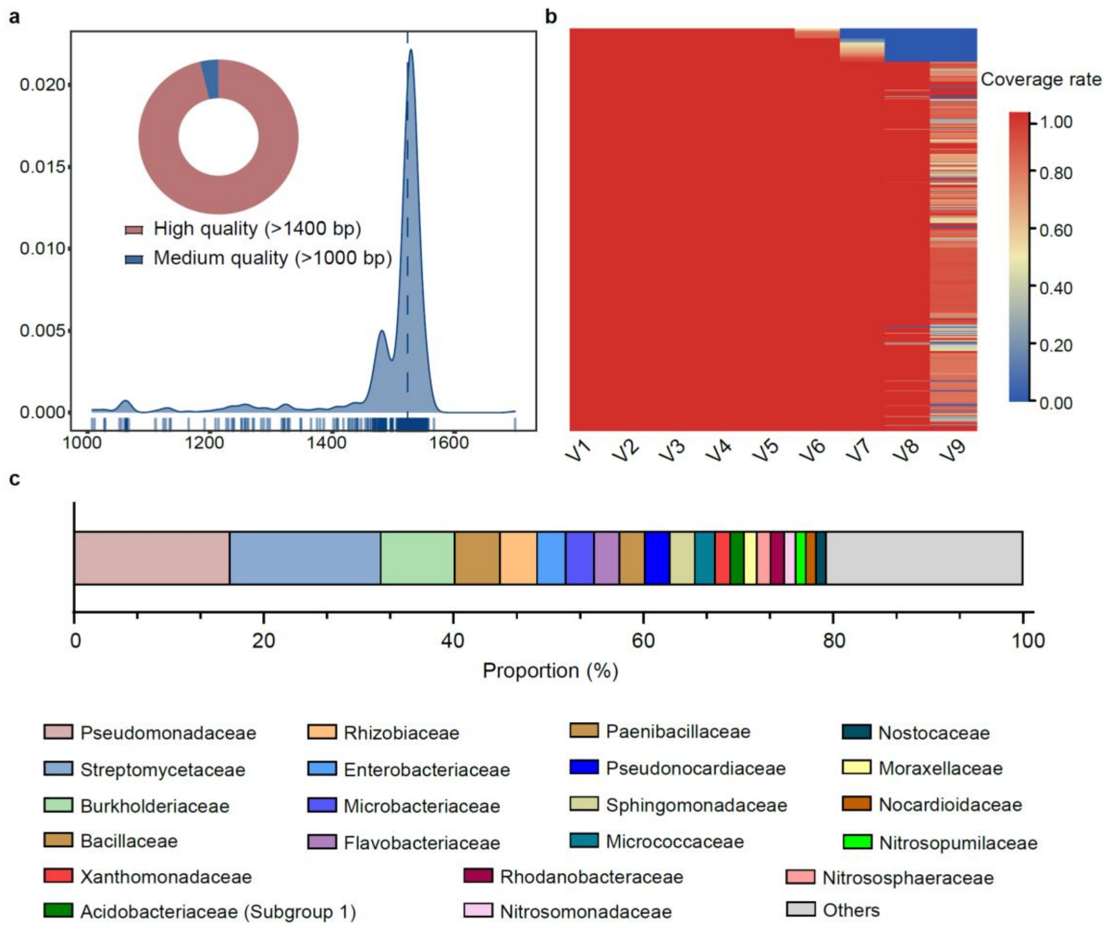

Supplementary Figure 18. Overview of the SCP v1.0_16S database. a, The quality of 16S sequences of cobamide-producing microbes in SCP v1.0_16S database. **b,** The coverage rates for different variable regions (V1-V9) of 16S rRNA gene sequences from the SCP v1.0_16S database. Colour gradations represent the coverage rate. **c,** The taxonomical composition (at family level) of cobamide-producing microbes in the SCP v1.0_16S database. Source Data are provided as a Source data file.

Supplementary Table 1 The adonis analysis of cobamide-producing microbes structure variation between various soil faunas.

	Earthworm	Potworm	Nematode	Oribatid mite	Collembolan
**Predatory mite**	0.13*	0.05*	0.04*	0.02*	0.11*
**Earthworm**		0.21*	0.19*	0.14*	0.065*
**Potworm**			0.01	0.05*	0.13*
**Nematode**				0.04*	0.11*
**Oribatid mite**					0.13*

Supplementary Table 2 The information of used primers of the DNA barcoding

Species	Primer	Reference
**Nematode**	5'segment of SSU	Floyd *et al*., 2002
**Potworm**	H3	Colgan *et al*., 1998
**Collembolan/ earthworm**	COI	Orgiazzi *et al*., 2015
**Mite**	28S rDNA D3-1	Fu Rong-Shu *et al.,* 2006

Reference:

1. Kriventseva, E.V., Kuznetsov, D., Tegenfeldt, F., et al. (2019). OrthoDB v10: sampling the diversity of animal, plant, fungal, protist, bacterial and viral genomes for evolutionary and functional annotations of orthologs. Nucleic Acids Research ***47***: D807-d811, 10.1093/nar/gky1053.
2. Ma, B., Lu, C., Wang, Y., et al. (2023). A genomic catalogue of soil microbiomes boosts mining of biodiversity and genetic resources. Nature Communications ***14***: 7318, 10.1038/s41467-023-43000-z.
3. Bolger, A.M., Lohse, M., and Usadel, B. (2014). Trimmomatic: a flexible trimmer for Illumina sequence data. Bioinformatics ***30***: 2114-2120, 10.1093/bioinformatics/btu170.
4. Langmead, B., and Salzberg, S.L. (2012). Fast gapped-read alignment with Bowtie 2. Nature Methods ***9***: 357-359, 10.1038/nmeth.1923.
5. Li, H., Handsaker, B., Wysoker, A., et al. (2009). The Sequence Alignment/Map

format and SAMtools. Bioinformatics ***25***: 2078-2079, 10.1093/bioinformatics/btp352.

6. Alneberg, J., Bjarnason, B.S., de Bruijn, I., et al. (2014). Binning metagenomic contigs by coverage and composition. Nature Methods ***11***: 1144-1146, 10.1038/nmeth.3103.
7. Wu, Y.W., Simmons, B.A., and Singer, S.W. (2016). MaxBin 2.0: an automated binning algorithm to recover genomes from multiple metagenomic datasets. Bioinformatics ***32***: 605-607, 10.1093/bioinformatics/btv638.
8. Kang, D.D., Li, F., Kirton, E., et al. (2019). MetaBAT 2: an adaptive binning algorithm for robust and efficient genome reconstruction from metagenome assemblies. PeerJ *7*: e7359, 10.7717/peerj.7359.
9. Parks, D.H., Imelfort, M., Skennerton, C.T., et al. (2015). CheckM: assessing the quality of microbial genomes recovered from isolates, single cells, and metagenomes. Genome Research ***25***: 1043-1055, 10.1101/gr.186072.114.
10. Wang, J., Zhu, Y.-G., Tiedje, J.M., and Ge, Y. (2024). Global biogeography and ecological implications of cobamide-producing prokaryotes. ISME Journal: Multidisciplinary Journal of Microbial Ecology ***18***: 1-12, 10.1093/ismejo/wrae009.
11. Kanehisa, M., Sato, Y., Kawashima, M., et al. (2016). KEGG as a reference resource for gene and protein annotation. Nucleic Acids Research ***44***: D457-462, 10.1093/nar/gkv1070.
12. Haft, D.H., Selengut, J.D., Richter, R.A., et al. (2013). TIGRFAMs and Genome Properties in 2013. Nucleic Acids Research ***41***: D387-395, 10.1093/nar/gks1234.
13. Finn, R.D., Coggill, P., Eberhardt, R.Y., et al. (2016). The Pfam protein families database: towards a more sustainable future. Nucleic Acids Research ***44***: D279-285, 10.1093/nar/gkv1344.
14. Johnson, L.S., Eddy, S.R., and Portugaly, E. (2010). Hidden Markov model speed heuristic and iterative HMM search procedure. BMC Bioinformatics ***11***: 431, 10.1186/1471-2105-11-431.

15. Lu, X., Heal, K.R., Ingalls, A.E., et al. (2020). Metagenomic and chemical characterization of soil cobalamin production. The ISME Journal ***14***: 53-66, 10.1038/s41396-019-0502-0.
16. Doxey, A.C., Kurtz, D.A., Lynch, M.D., et al. (2015). Aquatic metagenomes implicate Thaumarchaeota in global cobalamin production. The ISME Journal ***9***: 461-471, 10.1038/ismej.2014.142.
17. Shelton, A.N., Seth, E.C., Mok, K.C., et al. (2019). Uneven distribution of cobamide biosynthesis and dependence in bacteria predicted by comparative genomics. The ISME Journal ***13***: 789-804, 10.1038/s41396-018-0304-9.
18. Chaumeil, P.A., Mussig, A.J., Hugenholtz, P., and Parks, D.H. (2019). GTDB-Tk: a toolkit to classify genomes with the Genome Taxonomy Database. Bioinformatics ***36***: 1925-1927, 10.1093/bioinformatics/btz848.
19. Zhang, Z., Zhang, Q., Wang, T., et al. (2022). Assessment of global health risk of antibiotic resistance genes. Nature Communications ***13***: 1553, 10.1038/s41467-022-29283-8.
20. Lagesen, K., Hallin, P., Rødland, E.A., et al. (2007). RNAmmer: consistent and rapid annotation of ribosomal RNA genes. Nucleic Acids Research ***35***: 3100-3108, 10.1093/nar/gkm160.
21. Olsen, G.J., Overbeek, R., Larsen, N., et al. (1992). The Ribosomal Database Project. Nucleic Acids Research ***20 Suppl***: 2199-2200, 10.1093/nar/20.suppl.2199.
22. Shen, W., Sipos, B., and Zhao, L. (2024). SeqKit2: A Swiss army knife for sequence and alignment processing. Imeta ***3***: e191, 10.1002/imt2.191.
23. Schmieder, R., and Edwards, R. (2011). Quality control and preprocessing of metagenomic datasets. Bioinformatics ***27***: 863-864, 10.1093/bioinformatics/btr026.
24. Cock, P.J., Antao, T., Chang, J.T., et al. (2009). Biopython: freely available Python tools for computational molecular biology and bioinformatics. Bioinformatics ***25***: 1422-1423, 10.1093/bioinformatics/btp163.
25. Chen, Y., Ye, W., Zhang, Y., and Xu, Y. (2015). High speed BLASTN: an

accelerated MegaBLAST search tool. Nucleic Acids Research ***43***: 7762-7768, 10.1093/nar/gkv784.

26. Zhu, D., Delgado-Baquerizo, M., Ding, J., et al. (2021). Trophic level drives the host microbiome of soil invertebrates at a continental scale. Microbiome ***9***: 189, 10.1186/s40168-021-01144-4.

27. Bardgett, R.D., and van der Putten, W.H. (2014). Belowground biodiversity and ecosystem functioning. Nature ***515***: 505-511, 10.1038/nature13855.

28. Abramoff, M., Magalhães, P., and Ram, S.J. (2003). Image Processing with ImageJ. Biophotonics International ***11***: 36-42.
