# Supplementary material for: Cobamide-producing microbes as a model for understanding general nutritional interdependencies in soil food webs
Source: Nat Commun. 2026 Jan 13;17:1533. doi: 10.1038/s41467-025-68255-6 (PMC12891699; doi:10.1038/s41467-025-68255-6)
Supplement: Supplementary file 2 — Descriptions of Additional Supplementary Files [file 41467_2025_68255_MOESM2_ESM.pdf]

Description of Additional Supplementary Files

File Name: Supplementary Data 1
Description: Metadata of 7,874 high-quality metagenome-assembled genomes reconstructed from soil metagenomes.

File Name: Supplementary Data 2
Description: Metadata of 2,727 soil metagenomic samples used in this study.

File Name: Supplementary Data 3
Description: Identification of 4,341 cobamide-producing metagenome-assembled genomes.

File Name: Supplementary Data 4
Description: Soil cobamide producers detected in the gut microbiomes of six faunal groups.

File Name: Supplementary Data 5
Description: The HMM annotation file of *Bacillus megaterium* ATCC 14581 and *Streptomyces violaceus* NBC_00450.

File Name: Supplementary Data 6
Description: Summary of statistical tests for Figure 5b–f.

File Name: Supplementary Data 7
Description: Metadata of 20,933 16S rRNA amplicon sequencing datasets from the gut microbiomes used in this study.

File Name: Supplementary Data 8
Description: Calculation of soil contact degree of each animal.

File Name: Supplementary Data 9
Description: Metadata of 132 soil faunal reference genomes used in this study.

File Name: Supplementary Data 10
Description: Metadata of 5,363 soil reference genomes from NCBI used in this study.

File Name: Supplementary Data 11
Description: Classification method of cobamide producer in metagenome-assembled genomes.

File Name: Supplementary Data 12
Description: Sampling imformation of soil faunas across China.
