# Supplementary material for: Cobamide-producing microbes as a model for understanding general nutritional interdependencies in soil food webs
Source: Nat Commun. 2026 Jan 13;17:1533. doi: 10.1038/s41467-025-68255-6 (PMC12891699; doi:10.1038/s41467-025-68255-6)
Supplement: Supplementary file 15 — Reporting Summary [file 41467_2025_68255_MOESM15_ESM.pdf]

nature portfolio

Corresponding author(s): Haifeng Qian

Last updated by author(s): Dec 17, 2025

Reporting Summary

Nature Portfolio wishes to improve the reproducibility of the work that we publish. This form provides structure for consistency and transparency in reporting. For further information on Nature Portfolio policies, see our Editorial Policies and the Editorial Policy Checklist.

Statistics

For all statistical analyses, confirm that the following items are present in the figure legend, table legend, main text, or Methods section.

n/a	Confirmed	
☐	☒	The exact sample size (*n*) for each experimental group/condition, given as a discrete number and unit of measurement
☐	☒	A statement on whether measurements were taken from distinct samples or whether the same sample was measured repeatedly
☐	☒	The statistical test(s) used AND whether they are one- or two-sided<br>*Only common tests should be described solely by name; describe more complex techniques in the Methods section.*
☒	☐	A description of all covariates tested
☐	☒	A description of any assumptions or corrections, such as tests of normality and adjustment for multiple comparisons
☐	☒	A full description of the statistical parameters including central tendency (e.g. means) or other basic estimates (e.g. regression coefficient) AND variation (e.g. standard deviation) or associated estimates of uncertainty (e.g. confidence intervals)
☐	☒	For null hypothesis testing, the test statistic (e.g. *F*, *t*, *r*) with confidence intervals, effect sizes, degrees of freedom and *P* value noted<br>*Give P values as exact values whenever suitable.*
☒	☐	For Bayesian analysis, information on the choice of priors and Markov chain Monte Carlo settings
☒	☐	For hierarchical and complex designs, identification of the appropriate level for tests and full reporting of outcomes
☐	☒	Estimates of effect sizes (e.g. Cohen's *d*, Pearson's *r*), indicating how they were calculated

Our web collection on statistics for biologists contains articles on many of the points above.

Software and code

Policy information about availability of computer code

Data collection	Soil metagenomic assemblies were reconstructed using MEGAHIT v1.2.9, integrated with MetaWRAP assembly and binning commands. The quality of metagenome-assembled genomes (MAGs) was assessed using CheckM v1.0.11. Ribosomal RNA (rRNA) and transfer RNA (tRNA) genes within the MAGs were identified using the nhmmer function from HMMER 3 (as part of Barrnap v0.9) and tRNAscan-SE v2.0.9, respectively.A cobamide biosynthetic gene database was constructed using Hidden Markov Models (HMMs) based on the Kyoto Encyclopedia of Genes and Genomes (KEGG), TIGRFAM, and PFAM databases. The HMM search v3.3.2 tool was employed to identify cobamide biosynthetic genes within the predicted proteins of each MAG, with an E-value cut-off of 1×10^{-6}.The Soil Cobamide Producer Database v1.0_16S was established using Barrnap (v0.9) with default parameters, VSEARCH with default settings based on the RDP Gold database, SeqKit2, Prinseq (executed via the command prinseq-lite.pl), and BLAST searches against the SILVA 138.1 SSU Ref_NR99 database. Bacterial 16S rRNA gene sequences were analyzed using Quantitative Insights Into Microbial Ecology (QIIME, version 1.9.1) following the platform's standard guidelines.The transcriptomes of soil fauna were sequenced on the Illumina NovaSeq 6000 platform (Meiji, Shanghai, China). Functional genes within cobamide-producing MAGs were annotated using the KEGG database via DIAMOND v2.1.4.
Data analysis	The data are presented as the means ± standard errors of the means. The Kolmogorov–Smirnov test was used to assess the normality of the data distribution. The Brown–Forsythe test and F test were conducted to examine the homogeneity of variances for two groups or more, respectively. When the data were normally distributed and the variances were homogeneous, one-way analysis of variance (ANOVA) with Bartlett's test was used for multiple-group comparisons, and Student's one-sided t test was employed for between-group comparisons. If the data did not meet these assumptions, the Mann–Whitney and Kruskal–Wallis tests were applied for pairwise comparisons of two or more groups. Power analyses for t tests and ANOVA were conducted to ensure adequate sample sizes for detecting significant effects. All the statistical analyses and graphical visualizations were performed via GraphPad Prism 9 and IBM SPSS Statistics (v.20.0.0). Principal coordinate analyses and Adonis tests were performed via the "vegan" (v.2.6-2) and "pairwiseAdonis" (v.0.4.1) packages in R (v.3.6.3), respectively, and heatmaps were generated via TBtools (v.1.082). Correlations within the relative abundance of the prokaryotic species network were

calculated for each group via Pearson's correlation (P < 0.05) in R, and the calculation of network topology data was performed via Gephi (v.10.0.3). Venn diagrams were created via EVenn software (http://www.ehbio.com/test/venn). Data figures were constructed via GraphPad Prism 9 and the "ggplot2" (v.3.5.0) package in R (v.3.6.3), and Adobe Illustrator 2020 was used for figure formatting. All schematic diagrams and art elements were designed via BioRender (https://www.biorender.com). Maximum-likelihood phylogenetic trees were constructed via ITOL (https://itol.embl.de/upload.cgi). Gut microbial function was predicted via PICRUSt2 (www.majorbio.com). Maps used in Figures 3a, 6a, and Supplementary Figures 2(a), 3, and 4(a) were generated in Python using the 'Basemap' package (v.1.3.6) with publicly available coastline and land-mass data from the Natural Earth dataset (https://www.naturalearthdata.com), which is released under the public domain. The use of Basemap and Natural Earth data complies with their respective terms of use and requires no additional licensing for publication.

For manuscripts utilizing custom algorithms or software that are central to the research but not yet described in published literature, software must be made available to editors and reviewers. We strongly encourage code deposition in a community repository (e.g. GitHub). See the Nature Portfolio guidelines for submitting code & software for further information.

Data

Policy information about availability of data

All manuscripts must include a data availability statement. This statement should provide the following information, where applicable:

- Accession codes, unique identifiers, or web links for publicly available datasets
- A description of any restrictions on data availability
- For clinical datasets or third party data, please ensure that the statement adheres to our policy

The raw 16S rRNA gene amplicon sequencing data generated in this study have been deposited in the NCBI Sequence Read Archive under BioProject accession codes PRJNA916760. The genomes of Streptomyces violaceus NBC_00450 and Bacillus megaterium ATCC 14581 are available in public repositories, and their accession numbers are SAMN24016225 and SAMN15857556, respectively. The raw metagenomic datasets generated in this study have been deposited in public repositories, and the corresponding accession numbers are provided in Supplementary Data 2. Accession numbers for the publicly available 16S rRNA amplicon datasets analyzed here are listed in Supplementary Data 7. All soil faunal reference genomes included in this study are publicly accessible, with detailed accession information given in Supplementary Data 9. In addition, the soil bacterial genomes examined in this work are available from public repositories, and their accession numbers are summarized in Supplementary Data 10. The transcriptome dataset generated from Enchytraeus in this study has been deposited in the NCBI SRA under the BioProject accession PRJNA1373487. The SMAG catalog generated by Ma et al. 26 is publicly accessible via the Zenodo repository at https://doi.org/10.5281/zenodo.7341719. All other processed data supporting the findings of this study are provided in Supplementary Information. Source data are provided with this paper.

Research involving human participants, their data, or biological material

Policy information about studies with human participants or human data. See also policy information about sex, gender (identity/presentation), and sexual orientation and race, ethnicity and racism.

Reporting on sex and gender	No human research were involved in this study.
Reporting on race, ethnicity, or other socially relevant groupings	No human research were involved in this study.
Population characteristics	No human research were involved in this study.
Recruitment	No human research were involved in this study.
Ethics oversight	No human research were involved in this study.

Note that full information on the approval of the study protocol must also be provided in the manuscript.

Field-specific reporting

Please select the one below that is the best fit for your research. If you are not sure, read the appropriate sections before making your selection.

☐ Life sciences ☐ Behavioural & social sciences ☒ Ecological, evolutionary & environmental sciences

For a reference copy of the document with all sections, see nature.com/documents/nr-reporting-summary-flat.pdf

Ecological, evolutionary & environmental sciences study design

All studies must disclose on these points even when the disclosure is negative.

Study description	We developed the Soil Cobamide Producer Database (covering both metagenomic and 16S rRNA amplicon data) by integrating over 48,000 metagenomic and genomic datasets collected from 1,123 sampling sites across diverse global terrestrial ecosystems. Our analysis highlighted the critical role of cobamide-producing microbes in maintaining soil health through nutritional interdependencies within the soil food web. This was demonstrated using a combination of large-scale field studies, microcosm experiments, and artificial colonization trials.
Research sample	Metadata: A total of 132 representative genomes of soil fauna were obtained from the National Center for Biotechnology

Research sample	Information (NCBI) Genome Browser. Additionally, 2,727 soil metagenomes from 24 countries across six continents were retrieved from the European Nucleotide Archive. Furthermore, 5,363 representative bacterial genomes and 40,039 high-quality metagenome-assembled genomes (MAGs) derived from soil were collected from NCBI and the European Nucleotide Archive. In addition, 20,933 16S rRNA amplicon sequencing datasets from the gut microbiomes of 19 mammalian species (including humans), 7 bird species, 7 insect species, 1 annelid species, and 2 amphibian and reptile species were sourced from 150 locations across 31 countries on six continents. These datasets were retrieved from Google Scholar and the Sequence Read Archive (SRA) database of NCBI. Large-scale field experiments: Representative soil fauna from the soil food web were collected, including 238 collembolan, 60 nematode, 62 potworm, 146 oribatid mite, 122 predatory mite, and 50 earthworm samples from China. Microcosmic and artificial colonization experiments: The soil fauna Enchytraeus crypticus, a key species in soil ecosystems due to its dominance in both biomass and abundance (ranging from 10^2 to 10^5 individuals/m²), was selected as the model organism for these experiments.<br>All data analyses were based on publicly available datasets retrieved from the NCBI Genome Browser, the European Nucleotide Archive, and the Sequence Read Archive, ensuring reproducibility through open access and standardized processing pipelines. Large-scale field sampling and microcosmic experiments were independently replicated at least three times to confirm consistency. In all cases, the experimental results were successfully reproduced, and no failed replication attempts were recorded. Metadata collection and bioinformatic analyses were verified by multiple researchers using the same computational workflows to ensure analytical reproducibility.
Sampling strategy	We selected these metadata for their extensive coverage of sampling sites, species, and habitats, which were essential for constructing the comprehensive Soil Cobamide Producer Database. Additionally, soil fauna samples were collected across China from soils with diverse land-use types, ensuring a representative dataset to investigate their distribution and functional roles within the soil food web.
Data collection	The metadata samples were downloaded by QZ from the European Nucleotide Archive using the IBM Aspera Data Transfers service. Field samples were collected by DZ from six sites across China, spanning latitudes from 24.9°N to 41.7°N and longitudes from 102.95° E to 123.72°E, thereby covering most of the country's climatic zones. Laboratory samples were collected by QZ. Detailed procedures for sample collection are provided in the Methods section.
Timing and spatial scale	Representative genomes of soil faunas, large-scale metaproteomes, representative bacterial genomes, and 16S rRNA amplicon sequencing data were downloaded on July 20, 2023, January 20, 2022, March 16, 2022, and February 10, 2024, respectively. Field sampling was conducted across six sites in China between October and November 2017, covering latitudes from 24.9°N to 41.7°N and longitudes from 102.95°E to 123.72°E.
Data exclusions	There was no data exclusion in this analysis.
Reproducibility	All scripts and codes for machine learning, visualization, bioinformatics, and statistical analyses related to this study are available online at https://github.com/QiZhang11/SCP-as-a-model-for-nutritional-interdependencies-in-soil-food-webs.
Randomization	Not relevant to this study, as all the data we used were public.
Blinding	Not relevant to this study, as all the data we used were public.

Did the study involve field work? ☐ Yes ☒ No

Reporting for specific materials, systems and methods

We require information from authors about some types of materials, experimental systems and methods used in many studies. Here, indicate whether each material, system or method listed is relevant to your study. If you are not sure if a list item applies to your research, read the appropriate section before selecting a response.

Materials & experimental systems

n/a	Involved in the study
☒	☐ Antibodies
☒	☐ Eukaryotic cell lines
☒	☐ Palaeontology and archaeology
☐	☒ Animals and other organisms
☒	☐ Clinical data
☒	☐ Dual use research of concern
☒	☐ Plants

Methods

n/a	Involved in the study
☒	☐ ChIP-seq
☒	☐ Flow cytometry
☒	☐ MRI-based neuroimaging

Animals and other research organisms

Policy information about studies involving animals; ARRIVE guidelines recommended for reporting animal research, and Sex and Gender in Research

Laboratory animals	The soil animals used in this study, Enchytraeus crypticus (33–40 days old), is a soil invertebrate belonging to the phylum Annelida, class Clitellata, order Oligochaeta and family Enchytraeidae.

Wild animals	This study did not involve wild animals.
Reporting on sex	Enchytraeids are generally obligatory amphimictic hermaphrodites.
Field-collected samples	Field sampling was conducted across six sites in China between October and November 2017, spanning latitudes from 24.9° to 41.7° N and longitudes from 102.95° to 123.72° E, and the detailed field information was available in Dataset S10.
Ethics oversight	We performed all experiments according to the Organization for Economic Cooperation and Development (OECD) guidelines (OECD, 2004).

Note that full information on the approval of the study protocol must also be provided in the manuscript.

Plants

Seed stocks	This study did not involve plants.
Novel plant genotypes	This study did not involve plants.
Authentication	This study did not involve plants.
